# Food Insecurity Prevalence and Risk Factors at a Large Academic Medical Center in Michigan

**DOI:** 10.1001/jamanetworkopen.2024.3723

**Published:** 2024-03-26

**Authors:** Cindy W. Leung, Minal R. Patel, Markell Miller, Eileen Spring, Zixi Wang, Julia A. Wolfson, Alicia J. Cohen, Michele Heisler, Wei Hao

**Affiliations:** 1 Department of Nutrition, Harvard T.H. Chan School of Public Health, Boston, Massachusetts; 2 Department of Health Behavior and Health Education, University of Michigan School of Public Health, Ann Arbor; 3 Food Gatherers, Ann Arbor, Michigan; 4 Department of Biostatistics, University of Michigan School of Public Health, Ann Arbor; 5 Department of International Health, Johns Hopkins Bloomberg School of Public Health, Baltimore, Maryland; 6 Department of Health Policy and Management, Johns Hopkins Bloomberg School of Public Health, Baltimore, Maryland; 7 Center for Innovation in Long Term Services and Supports, VA Providence Healthcare System, Providence, Rhode Island; 8 Department of Family Medicine, Alpert Medical School of Brown University, Providence, Rhode Island; 9 Department of Health Services, Policy, and Practice, Brown University School of Public Health Providence, Rhode Island; 10 Department of Internal Medicine, University of Michigan Medical School, Ann Arbor

## Abstract

**Importance:**

Health care systems are increasingly adopting methods to screen for and integrate food insecurity and other social risk factors into electronic health records. However, there remain knowledge gaps regarding the cumulative burden of food insecurity in large clinical settings, which patients are most at risk, and the extent to which patients are interested in social assistance through their health care system.

**Objective:**

To evaluate the 5-year prevalence and associated risk factors of food insecurity among adult primary care patients, and to examine factors associated with patients’ interest in social assistance among those with food insecurity.

**Design, Setting, and Participants:**

This cross-sectional analysis of a retrospective cohort study took place at a tertiary care academic medical center (encompassing 20 primary care clinics) in Michigan. Participants included adult patients who completed screening for social risk factors between August 1, 2017, and August 1, 2022. Data analysis was performed from November 2022 to June 2023.

**Exposure:**

Food insecurity was assessed using the Hunger Vital Sign.

**Main Outcomes and Measures:**

The primary outcome was patients’ interest in social assistance, and associated factors were examined using multivariate logistic regression models, adjusting for patients’ demographic and health characteristics.

**Results:**

Over the 5-year period, 106 087 adult primary care patients (mean [SD] age, 52.9 [17.9] years; 61 343 women [57.8%]) completed the standardized social risk factors questionnaire and were included in the analysis. The overall prevalence of food insecurity was 4.2% (4498 patients), with monthly trends ranging from 1.5% (70 positive screens) in August 2018 to 5.0% (193 positive screens) in June 2022. Food insecurity was significantly higher among patients who were younger, female, non-Hispanic Black or Hispanic, unmarried or unpartnered, and with public health insurance. Food insecurity was significantly associated with a higher cumulative burden of social needs, including social isolation, medical care insecurity, medication nonadherence, housing instability, and lack of transportation. Only 20.6% of patients with food insecurity (927 patients) expressed interest in social assistance. Factors associated with interest in social assistance including being non-Hispanic Black, unmarried or unpartnered, a current smoker, and having a higher burden of other social needs.

**Conclusions and Relevance:**

In this retrospective cohort study, the overall prevalence of food insecurity was 4.2%, of whom approximately 1 in 5 patients with food insecurity expressed interest in assistance. This study highlights ongoing challenges in ensuring all patients complete routine social determinants of health screening and gaps in patients’ interest in assistance for food insecurity and other social needs through their health care system.

## Key Points

**Question:**

What factors are associated with food insecurity and interest in social assistance among adult primary care patients?

**Findings:**

In this 5-year cohort study of 106 087 patients, the overall prevalence of food insecurity was 4.2%, and 20.6% of those patients expressed interest in assistance. Patients identifying as non-Hispanic Black, unmarried or unpartnered, and having additional unmet social needs were more likely than patients with other demographic profiles to be food insecure and express interest in assistance.

**Meaning:**

Challenges remain in ensuring all patients are screened and patients with food insecurity are supported in expressing interest in social assistance through their health care system.

## Teaser

This cohort study evaluates the 5-year prevalence and associated risk factors of food insecurity among adult primary care patients in Michigan and examines factors associated with patients’ interest in social assistance among those with food insecurity.

## Introduction

Food insecurity, a condition of limited access to healthy food, is an adverse social risk factor affecting 33.8 million individuals in the US in 2021.^1^ Food insecurity has long-term health implications, including cardiovascular disease and diabetes,^2^ and contributes to $77.5 billion in excess health care expenditures annually.^3^ The growing recognition that food insecurity and other social risk factors exert powerful influences on health has led to widespread efforts to screen, document, and address social determinants of health (SDH) in clinical settings. The National Academy of Medicine, Centers for Medicare & Medicaid Services, and several other health organizations have issued recommendations to screen for and address SDH in clinical settings.^4,5,6,7^ In 2022, The Joint Commission, the accreditation organization for more than 22 000 health care organizations and programs in the US, has required their hospitals and clinics to assess SDH among patients and provide them with relevant resources.^8^ Primary care clinics have been identified as ideal settings to screen for SDH because they provide opportunities for regular patient contact and preventive care.^9^ Research thus far indicates that both patients and practitioners find screening for food insecurity and other SDH in clinical settings highly acceptable and important by patients and practitioners.^10,11,12,13,14,15^

In August 2017, Michigan Medicine began routinely screening primary care patients for food insecurity and other SDH as part of the Michigan State Innovation Model Patient-Centered Medical Home Initiative.^16^ The initiative’s screening tool is modeled after established screeners^17^ and comprises questions assessing a range of health-related social risk factors, including food insecurity, housing instability, transportation needs, and health care affordability. Although many large health care systems are increasingly adopting methods to screen for and integrate SDH data into electronic health records (EHRs), these methods need to be evaluated to understand the cumulative burden of food insecurity and other SDH in the patient population, and to identify improvement areas to optimize health care–based interventions to reach patients with food insecurity.^9^ At Michigan Medicine, the screening tool includes an additional question about whether the patient would like to request assistance from the central office of trained social workers to provide referrals to community resources. Current research^18,19^ suggests there is a gap between patients who screen positive for social risk factors and those who seek assistance, which may be related to multiple reasons, including patients already engaging with existing resources or stigma associated with receiving assistance. Using a retrospective cohort design, we sought to (1) evaluate the 5-year prevalence and associated risk factors of food insecurity among adult primary care patients, and (2) examine factors associated with patients’ interest in assistance among those screening positive for food insecurity.

## Methods

### Study Participants

Michigan Medicine, a tertiary care academic medical center, is one of the largest health care systems in Michigan (the statewide prevalence of food insecurity is 11.4%)^1^ with more than 2.6 million annual patient visits. We conducted a cross-sectional analysis of a retrospective cohort study using data for all adults (aged ≥18 years) who completed SDH screening at a primary care patient encounter between August 2017 and August 2022 at a Michigan Medicine clinic. Primary care encounters included those that occurred at 8 affiliated outpatient general medicine clinics, 9 family medicine clinics, and 3 geriatrics medicine clinics across southeast Michigan. Both in-person and virtual encounters were included. For patients with multiple encounters during this 5-year period, encounters were prioritized if a patient ever screened positive for food insecurity. The most recent encounter was used for patients who screened positive for food insecurity more than once and for patients who consistently screened negative for food insecurity. This study was considered exempt by the University of Michigan institutional review board, and informed consent was not needed because the data were anonymous, in accordance with 45 CFR §46. This report follows the Strengthening the Reporting of Observational Studies in Epidemiology (STROBE) reporting guidelines for cohort studies.

### Food Insecurity and Other Social Risk Factors

In August 2017, Michigan Medicine began screening primary care patients for SDH through a standardized questionnaire. The questionnaire is meant to be administered at all new patient visits or annually during routine health maintenance examinations. The questionnaire is offered electronically to patients via their online patient portal 30 days or less in advance of their visit date. Patients who have not completed the electronic questionnaire before the visit are then prompted by the front desk to complete the paper questionnaire (or electronic questionnaire, if a tablet is available) at their visit. Paper responses are entered into the EHR by clinic staff.

Food insecurity is assessed using the 2-item Hunger Vital Sign, a validated measure developed specifically for clinical food insecurity screening.^20^ The Hunger Vital Sign is composed of the first 2 questions of the US Department of Agriculture’s Household Food Security Survey Module: “Within the past 12 months, you worried that your food would run out before you got money to buy more,” and “Within the past 12 months, the food you bought just didn’t last and you didn’t have money to get more.” Response options were initially presented as yes, no, or don’t know, although beginning March 2019, several clinics switched to the response options of often true, sometimes true, and never true. An affirmative response to either question is considered a positive food insecurity screen. These questions combined with the 3-category response have been shown to have 97% sensitivity and 93% specificity.^21^

In addition to screening for food insecurity, the standardized questionnaire assessed other social risk factors, including interpersonal violence; housing, transportation, and utility insecurities; unemployment; medication nonadherence; and social isolation (eTable 1 in Supplement 1). The number of unmet social needs was collapsed into 3 categories for analysis: 0, 1 to 2, and 3 or more. At the end of the questionnaire, patients were asked whether they wanted to be contacted by a trained social worker: “Do you want to get connected with resources for any of the above responses?” Responses to the questionnaire were documented in the EHR and linked to patients’ demographic and health data from the same encounter.

### Demographic and Health Data

Patient demographic and health data were extracted from the EHR and categorized for analysis, including age (18-34, 35-44, 45-54, 55-64, 65-74, or ≥75 years), sex assigned at birth (male or female), race and ethnicity (Hispanic, non-Hispanic Asian, non-Hispanic Black, non-Hispanic White, and non-Hispanic other, which includes American Indian and Alaska Native, Hawaiian or Pacific Islander, other nonspecified race, and multiracial), marital status (married or partnered, single, separated, divorced, or widowed), health insurance (private or public), smoking behavior (nonsmoker, current smoker, or former smoker), alcohol use (nondrinker, current drinker, or former drinker), and body mass index (BMI; calculated as weight in kilograms divided by height in meters squared; <25.0, 25.0-29.9, 30.0-34.9, and ≥35.0). BMI was not available for 4449 virtual or telephone encounters. Data on race and ethnicity were included in this study because of known disparities in food insecurity rates, largely due to structural factors. Overall missingness for demographic and health variables were 4.9% for race and ethnicity, 9.6% for marital status, 0.5% for health insurance, 1.3% for smoking behavior, 2.8% for alcohol use, and 6.4% for BMI.

### Statistical Analysis

Data analysis was performed from November 2022 to June 2023. First, we examined monthly trends in social needs screening and positive food insecurity rates across the health care system from August 1, 2017, through August 1, 2022. Next, we performed descriptive analyses to estimate the overall prevalence of food insecurity in the patient sample and differences in patients’ demographic and health behaviors by food insecurity using χ^2^ tests. We then examined the coexistence of other social needs by food insecurity using χ^2^ tests. Finally, we examined factors associated with patients’ interest in assistance among those who screened positive for food insecurity using multivariate logistic regression models, adjusting for patients’ demographic and health characteristics. Statistical tests were 2-sided, and significance was determined at *P* < *.*05. All statistical analyses were performed using SAS statistical software version 9.4 (SAS Institute).

## Results

The total sample was 403 515 patients, and 127 110 (31.5%) completed SDH screening over the 5-year period, with the screening rate ranging from 6% to 48% at individual clinics. After exclusions, 106 087 patients (mean [SD] age, 52.9 [17.9] years; 61 343 women [57.8%]) representing 194 717 total encounters were included in the analysis. The overall prevalence of positive food insecurity screens was 4.2% (4498 patients). The Figure shows the monthly count of patient screens that occurred after SDH screening was implemented and the monthly variability in the proportion of positive food insecurity. The lowest monthly prevalence of food insecurity was observed in August 2018 (1.5%; 70 positive screens), and the highest prevalence was observed in June 2022 (5.0%; 193 positive screens).

**Figure.**
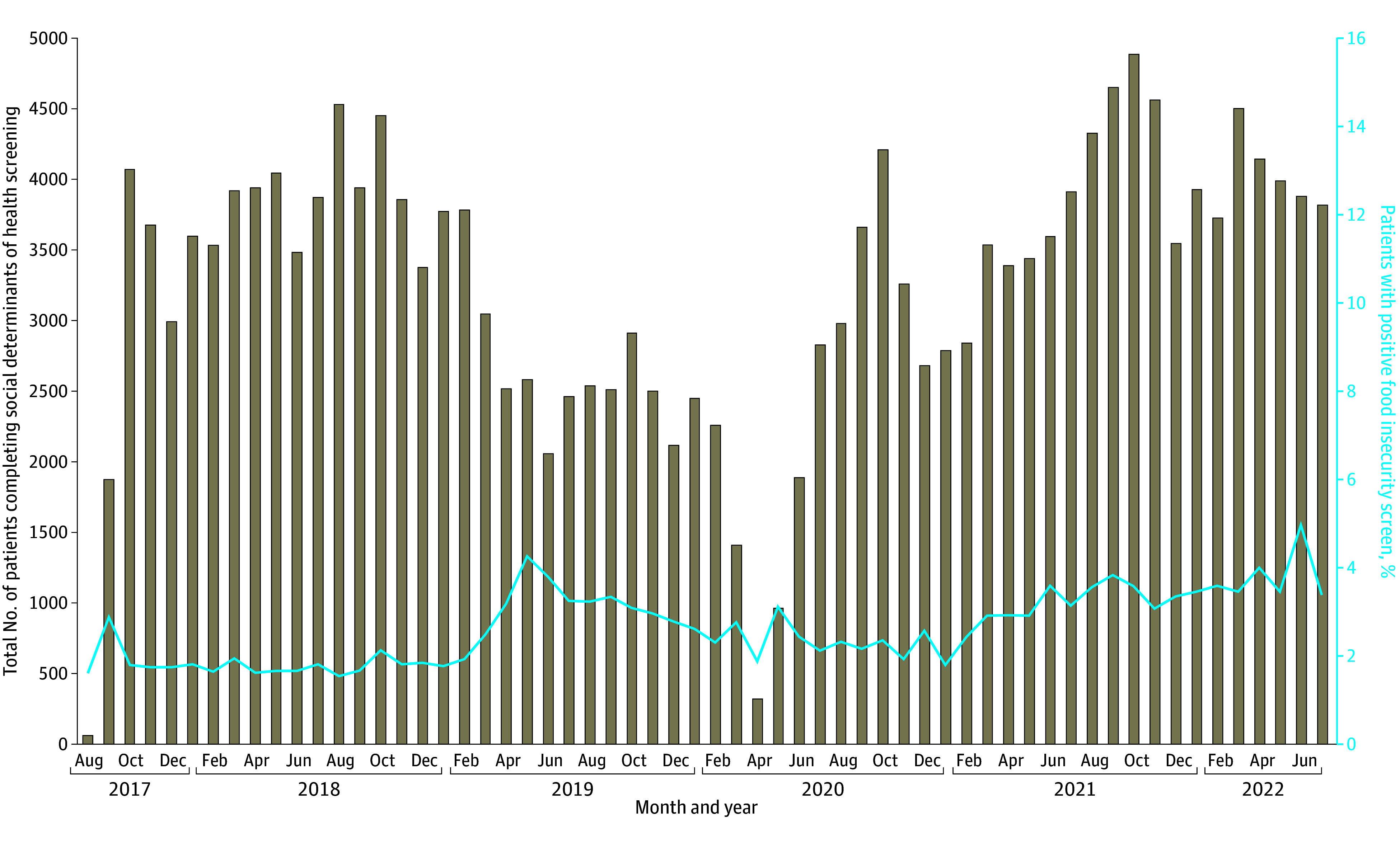
Michigan Medicine Adult Primary Care Patients Completing Social Determinants of Health Screening and Proportion With Positive Food Insecurity Screens, 2017-2022

There was significant variation in screening positive for food insecurity by patients’ demographic characteristics (Table 1). Food insecurity was significantly higher among younger (age 18-34 years, 1214 patients [6.0%]) vs older (age ≥65 years, 800 patients [2.6%]) patients and among female patients (3034 patients [4.9%]) vs male patients (1463 patients [3.3%]). By race and ethnicity, food insecurity was more prevalent among non-Hispanic Black patients (1080 patients [15.4%]) and Hispanic patients (219 patients [7.3%]) compared with non-Hispanic White patients (2725 patients [3.4%]). Differences in food insecurity were also observed by marital status (unmarried or unpartnered adults, 2570 patients [8.2%]; married or partnered adults, 1331 patients [2.1%]) and by insurance type (Medicaid, 88 patients [24.4%]; private health insurance, 3579 patients [4.1%]). There were also differences in screening positive for food insecurity by patients’ health characteristics. Patients who were current smokers (932 patients [14.3%]) were more likely to be food insecure than patients who were nonsmokers (2170 patients [3.1%]). Non–alcohol drinkers (1905 patients [6.1%]) were more likely to be food insecure than current drinkers (1868 patients [3.0%]). Patients with a BMI of 35.0 or higher (1249 patients [8.1%]) were more likely to be food insecure than patients with a BMI less than 25.0 (983 patients [3.1%]).

**Table 1.** Sociodemographic and Health Characteristics of Adult Primary Care Patients by Food Security Status

Characteristic	Patients, No. (%)	Food insecurity screen, No. of patients (row %) [column %]		*P* value^a^
		Positive	Negative	
Age range, y				
18-34	20 303 (19.1)	1214 (6.0) [27.0]	19 089 (94.0) [18.8]	<.001
35-44	15 455 (14.6)	741 (4.8) [16.5]	14 714 (95.2) [14.5]	
45-54	18 423 (17.4)	869 (4.7) [19.3]	17 554 (95.3) [17.3]	
55-64	20 099 (19.0)	874 (4.3) [19.4]	19 225 (95.7) [18.9]	
65-74	19 311 (18.2)	551 (2.9) [12.3]	18 760 (97.1) [18.5]	
≥75	12 496 (11.8)	249 (2.0) [5.5]	12 247 (98.0) [12.1]	
Sex				
Female	61 343 (57.8)	3034 (4.9) [67.5]	58 309 (95.1) [57.4]	<.001
Male	44 740 (42.2)	1463 (3.3) [32.5]	43 277 (96.7) [42.6]	
Race and ethnicity				
Hispanic	2988 (3.0)	219 (7.3) [5.0]	2769 (92.7) [2.9]	<.001
Non-Hispanic Asian	6358 (6.3)	113 (1.8) [2.6]	6245 (98.2) [6.5]	
Non-Hispanic Black	6993 (6.9)	1080 (15.4) [24.8]	5913 (84.6) [6.1]	
Non-Hispanic White	80 452 (79.8)	2725 (3.4) [62.5]	77 727 (96.6) [80.5]	
Other (non-Hispanic)^b^	4075 (4.0)	223 (5.5) [5.1]	3852 (94.5) [4.0]	
Marital status				
Married or significant other	62 037 (64.7)	1331 (2.1) [34.1]	60 706 (97.9) [66.0]	<.001
Single	25 734 (26.8)	1958 (7.6) [50.2]	23 776 (92.4) [25.8]	
Divorced or widowed	8166 (8.5)	612 (7.5) [15.7]	7554 (92.5) [8.2]	
Health insurance type				
Private	86 758 (82.2)	3579 (4.1) [80.6]	83 179 (95.9) [82.3]	.004
Medicare	18 291 (17.3)	772 (4.2) [17.4]	17 519 (95.8) [17.3]	
Medicaid	360 (0.3)	88 (24.4) [2.0]	272 (75.6) [0.3]	
Other public insurance	148 (0.1)	4 (2.7) [0.1]	144 (97.3) [0.1]	
Smoking				
Nonsmoker	69 852 (66.7)	2170 (3.1) [49.0]	67 682 (96.9) [67.5]	<.001
Current smoker	6540 (6.3)	932 (14.3) [21.0]	5608 (85.7) [5.6]	
Former smoker	28 321 (27.1)	1328 (4.7) [30.0]	26 993 (95.3) [26.9]	
Alcohol use				
Nondrinker	31 143 (30.2)	1905 (6.1) [43.7]	29 238 (93.9) [29.6]	<.001
Current drinker	62 092 (60.2)	1868 (3.0) [42.8]	60 224 (97.0) [61.0]	
Former drinker	9917 (9.6)	588 (5.9) [13.5]	9329 (94.1) [9.4]	
Body mass index^c^				
<25.0	31 300 (31.5)	983 (3.1) [23.2]	30 317 (96.9) [31.9]	<.001
25.0-29.9	32 955 (33.2)	1092 (3.3) [25.8]	31 863 (96.7) [33.5]	
30.0-34.9	19 539 (19.7)	906 (4.6) [21.4]	18 633 (95.4) [19.6]	
≥35.0	15 502 (15.6)	1249 (8.1) [29.5]	14 253 (91.9) [15.0]	

^a^ *P* values were estimated from χ^2^ tests.

^b^ Other race and ethnicity includes individuals identifying as American Indian and Alaska Native, Hawaiian or Pacific Islander, multiracial, or another unspecified race category.

^c^ Body mass index is calculated as weight in kilograms divided by height in meters squared.

Table 2 shows the coexistence of other social needs by patients’ food security status. Compared with patients with a negative food insecurity screen, patients with a positive food insecurity screen were more likely to endorse every social need, including housing instability, employment insecurity, utility insecurity, cost-related medication nonadherence, medical care insecurity, interpersonal violence (including physical, emotional, or sexual abuse), transportation insecurity, child care or elder care problems, and social isolation. Among patients with a positive food insecurity screen, the 5 most common social needs were social isolation (1679 patients [54.6%]), medical care insecurity (460 patients [27.6%]), cost-related medication nonadherence (1105 patients [25.4%]), housing instability (854 patients [19.7%]), and lack of transportation for work and daily living (612 patients [17.7%]). Furthermore, 47.2% of patients (2119 patients) with a positive food insecurity screen endorsed 1 or 2 other social needs, and 24.9% of patients (1116 patients) endorsed 3 or more other social needs.

**Table 2.** Prevalence of Other Unmet Social Needs of Adult Primary Care Patients by Food Security Status

Social risk factors	Patients, No. (%)			*P* value^b^
	Overall^a^	Food insecurity screen		
		Positive	Negative	
Individual social risk factors				
Housing insecurity (worried about not having stable housing)	1472 (1.4)	854 (19.7)	618 (0.6)	<.001
Employment insecurity (hard time finding work)	1988 (1.9)	657 (15.4)	1331 (1.3)	<.001
Utility insecurity (company shut off service)	962 (0.9)	428 (9.8)	534 (0.5)	<.001
Medical care insecurity (need help paying for medical care)	1226 (2.7)	460 (27.6)	766 (1.7)	<.001
Medication nonadherence (skipped medications to save money)	3117 (3.0)	1105 (25.4)	2012 (2.0)	<.001
Lack of transport kept you from medical care	1716 (1.6)	766 (17.4)	950 (1.0)	<.001
Lack of transport kept you from work or daily living activities	1232 (1.5)	612 (17.7)	620 (0.8)	<.001
Child care or elder care problems	1249 (1.4)	245 (6.7)	1004 (1.2)	<.001
Felt unsafe in your home or been afraid of someone close to you	768 (0.9)	242 (7.1)	526 (0.7)	<.001
Physical abuse	269 (0.3)	69 (2.0)	200 (0.3)	<.001
Emotional abuse (humiliated or emotionally abused)	1521 (1.9)	446 (13.3)	1075 (1.4)	<.001
Rape or sexual abuse	125 (0.2)	45 (1.3)	80 (0.1)	<.001
Social isolation	13 046 (18.1)	1679 (54.6)	11 367 (16.5)	<.001
No. of social risk factors (excluding food insecurity)				
0	86 430 (81.5)	1253 (27.9)	85 177 (83.9)	<.001
1-2	17 475 (16.5)	2119 (47.2)	15 356 (15.1)	
≥3	2088 (2.0)	1116 (24.9)	972 (1.0)	

^a^ Overall sample sizes for each social risk factor varied according to patients who chose to answer those questions.

^b^ *P* values were estimated from χ^2^ tests.

Among the 4498 patients with a positive food insecurity screen, 20.6% (927 patients) expressed interest in assistance for resources. Models estimating interest in assistance by patient characteristics are shown in Table 3. In model 1, patients identifying as non-Hispanic Black (adjusted odds ratio [aOR], 1.76; 95% CI, 1.44-2.17), single (aOR, 1.44; 95% CI, 1.15-1.79), or divorced or widowed (aOR, 2.02; 95% CI, 1.54-2.66) were more likely to show interest in assistance than patients identifying as non-Hispanic White or as married or partnered. Male patients (aOR, 0.75; 95% CI, 0.61-0.92) were less likely than female patients to show interest in assistance. In model 2 incorporating social need burden, those with 1 or 2 additional social needs (aOR, 3.76; 95% CI, 2.70-5.23) and 3 or more additional social needs (aOR, 12.33; 95% CI, 8.83-17.24) were more likely to show interest in assistance; most variables from model 1 remained significant. With the inclusion of additional health characteristics in model 3, current smokers (aOR, 1.39; 95% CI, 1.07-1.81) were more likely to show interest in assistance than nonsmokers, whereas current alcohol drinkers (aOR, 0.71; 95% CI, 0.57-0.89) were less likely to show interest in assistance than nondrinkers (eTable 2 in Supplement 1). Across the models, age, health insurance type, and BMI were not significantly associated with interest in assistance.

**Table 3.** Factors Associated With Interest in Assistance Among Adult Primary Care Patients Who Screened Positive for Food Insecurity

Characteristic	aOR (95% CI)^a^	
	Model 1	Model 2
Age range, y		
18-34	1 [Reference]	1 [Reference]
35-44	1.09 (0.82-1.45)	1.07 (0.79-1.46)
45-54	1.10 (0.84-1.45)	1.17 (0.87-1.56)
55-64	0.95 (0.72-1.27)	1.02 (0.75-1.39)
65-74	0.75 (0.53-1.08)	0.93 (0.64-1.35)
≥75	0.68 (0.42-1.09)	0.98 (0.59-1.63)
Sex		
Female	1 [Reference]	1 [Reference]
Male	0.75 (0.61-0.92)	0.76 (0.61-0.94)
Race and ethnicity		
Hispanic	1.16 (0.75-1.78)	1.23 (0.78-1.93)
Non-Hispanic Asian	1.27 (0.70-2.31)	1.35 (0.71-2.55)
Non-Hispanic Black	1.76 (1.44-2.17)	2.04 (1.64-2.55)
Non-Hispanic White	1 [Reference]	1 [Reference]
Other (non-Hispanic)^b^	1.27 (0.83-1.96)	1.27 (0.81-2.01)
Marital status		
Married or significant other	1 [Reference]	1 [Reference]
Single	1.44 (1.15-1.79)	1.16 (0.92-1.46)
Divorced or widowed	2.02 (1.54-2.66)	1.55 (1.16-2.08)
Health insurance type		
Private	1 [Reference]	1 [Reference]
Public	1.20 (0.94-1.54)	1.11 (0.85-1.45)
No. of social needs		
0	NA	1 [Reference]
1-2	NA	3.76 (2.70-5.23)
≥3	NA	12.33 (8.83-17.24)

Abbreviations: aOR, adjusted odds ratio; NA, not applicable.

^a^ Estimates are from multivariable logistic regression models.

^b^ Other race and ethnicity includes individuals identifying as American Indian and Alaska Native, Hawaiian or Pacific Islander, multiracial, or another unspecified race category.

## Discussion

In the 5-year period after implementing SDH screening at a large, tertiary care academic medical center, this cohort study found that the total prevalence of patients screening positive for food insecurity was 4.2%, ranging from 1.5% (August 2018) to 5.0% (June 2022). Notably, the monthly prevalence of positive food insecurity screens did not change substantially during the early months of the COVID-19 pandemic. This may be because of the smaller number of patients screened for SDH between April and June 2020, as well as systemic barriers to health care access among those at greatest risk of food insecurity.^22^

The overall prevalence of patient-reported food insecurity is lower than estimated rates for the state of Michigan (11.4%).^1^ One factor that may explain this is the self-selection of patients who seek primary care at Michigan Medicine. Patients were predominantly middle-aged or older, non-Hispanic White, married or partnered, and with private health insurance. Other institutional factors, such as the overall reputation of the health system, the perceived quality of care, and the number of specialty departments, may also attract patients with higher socioeconomic status, contributing to the lower levels of food insecurity in the patient population.^23^

Another factor is the low overall response rate to the SDH questionnaire. Only 31.5% of adult primary care patients had documented responses to the SDH questionnaire within the EHR, which may include patients who were not offered the questionnaire and patients who refused the questionnaire. At the clinic level, screening rates ranged from 6% to 48%. Individual clinic characteristics (eg, patient type, clinic location, number of physicians, staff knowledge on SDH screening, mode of administration, and other factors) may have led to variability in SDH screening rates. These results underscore the need for institutional efforts to identify clinic, practitioner, and patient characteristics that pose barriers to SDH screening. For example, some practitioners might feel challenged in administering the SDH questionnaire if they have time constraints or have a lack of knowledge about how to address specific social needs reported by the patient.^24^ Alternatively, patients might be declining to complete the questionnaire altogether because of shame, fear of stigmatization, or lack of interest. A prior Michigan-based study found that food insecurity screening was less common at urgent care or return visits, among Hispanic patients (compared with White patients), and among young and middle-aged adult patients (compared with older adult patients).^25^ Further research to understand specific barriers to SDH screening at each touchpoint is needed to inform efforts to improve practitioner and patient awareness on the importance of capturing SDH as part of patients’ routine health management.

Despite the lower overall prevalence of positive food insecurity screens, there was substantial variation, with food insecurity disproportionately affecting female patients, as well as those of younger age, non-Hispanic Black race and Hispanic ethnicity, those who reported current smoking behavior, and those who have higher BMIs. These differences by patients’ characteristics and health behaviors are consistent with studies using national survey data^1,26,27,28,29,30^ and studies conducted at other large, integrated health care systems.^14,31,32^ In the clinical context, these data, which are routinely captured in the EHR, can help clinicians integrate SDH discussions as part of overall health management for patients who may derive greater benefit from additional discussions around managing food resources and clinical interventions for food assistance. These data can also help alert clinicians to make modifications made to patients’ clinical care according to their food security status.

Our study also highlights the markedly high burden of additional social needs among adults with food insecurity. Nearly 3 of 4 adults screening positive for food insecurity had at least 1 other social need, with 1 in 4 adults screening positive for food insecurity having 3 or more other social needs. The most frequently endorsed concerns included social isolation, medical care insecurity, cost-related medication nonadherence, housing instability, and lack of transportation for daily activities. The large overlap between food insecurity and other social risk factors is corroborated by prior studies^33,34,35,36^ and points to the shared upstream contributors, including poverty and limited economic mobility, barriers to higher education, poor access to health care, systemic racism, and more. These highlight the importance of robust food, housing, economic, health, and social policies to dismantle structural factors underlying health inequities.^37^ At the patient level, prior research^36,38,39,40^ shows that the cumulative burden of unmet social needs can adversely affect health care utilization, health management behaviors, disease management, and overall mortality. Compounded with food insecurity—which already is an independent risk factor for poor diet and diet-sensitive chronic disease—these findings reinforce the need for health care–based interventions to support multiple social needs more holistically within the same individual and household.^41^

Addressing SDH through a health care system remains an evolving concept for both patients and practitioners. Existing models to address food insecurity in health care settings often include referrals to resources outside the health care system, such as a community food pantry and/or connections to other organizations that can help the patient apply for SNAP (Supplemental Nutrition Assistance Program) benefits and other food assistance programs.^42,43^ There is a growing research base to document health care–based interventions to address food insecurity and related social needs, but additional evidence is needed to identify best practices to close the gap between patient food insecurity and community resource engagement. In the present study, only 1 in 5 adults with food insecurity had interest in assistance, and likely far fewer successfully connected with the social worker, reached out to the provided resources, and ultimately received assistance.^44,45^ Prior studies^45,46^ have shown that patients may not remember opting out of requesting assistance on a questionnaire, may no longer be experiencing food insecurity at the time of the encounter, were overwhelmed with more urgent health concerns or other social needs, or felt their social needs were beyond the scope of what their health care practitioner could address. In a mixed methods study^19^ of adults with diabetes, additional barriers to engaging with social care assistance included stigma or prior negative experiences. These factors pose substantial challenges to addressing food insecurity and other social needs and, ultimately, promoting health, among patients at a large health care system. Further clinical and research efforts are warranted to improve current case management efforts, understand patients’ levels of resiliency and self-efficacy in managing social needs, identify barriers to requesting assistance across heterogeneous patient groups, and design individualized care plans that more holistically address patients’ SDH and precision health.

### Limitations

This study has several limitations. First, the retrospective cohort includes only adult patients who had a primary care encounter between August 2017 and August 2022. Patients who opted not to seek primary care at Michigan Medicine during this window or who were seen only at specialty clinics were not included in the sample. Second, because several clinics modified the response options to the Hunger Vital Sign questions in early 2019, this change may have contributed to the slight increase in positive food insecurity screens observed between March to May 2019. The overall prevalence of food insecurity may have been higher had all clinics adopted the higher-sensitivity, 3-category response since screening began in 2017.^47^ Another limitation is that the standardized patient questionnaire included a single broad question about the patient’s interest in assistance. Results show that patients were more likely to respond affirmatively to this question if they had multiple other unmet social needs (in addition to food insecurity), suggesting that food insecurity may not be the only or most pressing issue discussed in the encounter with the social worker. Qualitative studies are needed to provide additional context to patients’ responses to the screening questions, including factors contributing to their desire to request assistance and the extent to which food insecurity and other social needs were addressed during the encounter with the trained social worker. In addition, clinic variability in screening rates deserves further study. Information is not available on how each individual clinic implemented SDH screening; however, Michigan Medicine has made multiple efforts to streamline SDH screening protocols since 2017 and to expand SDH screening to specialty clinics in 2023. Future research may want to explore individual clinic SDH screening rates further, as well as identify clinic-specific strategies to increase response rates, on top of existing efforts at the level of the health care system. In addition, the results of this study may not be generalizable to health care systems in other states, geographic regions, or with different catchment areas.

## Conclusions

In this retrospective cohort study, the overall prevalence of food insecurity was 4.2%, of which approximately 1 in 5 patients with food insecurity expressed interest in assistance. Although efforts to screen and address food insecurity and other social risk factors in health care systems have grown substantially in the last decade, there are still major challenges around the implementation of SDH screening at a large health care system. Further research is needed to identify strategies to increase screening rates within clinics and across the health care system, to improve patient-practitioner communication around social risk factors, and to better provide social assistance to patients tailored to each of their social needs.

## Notes

Supplement 1. **eTable 1. **Domains and Questions Included in the Social Determinants of Health Screening at Michigan Medicine **eTable 2.** Demographic, Health, and Social Predictors of Interest in Assistance Among Adult Primary Care Patients Who Screened Positive for Food Insecurity

Supplement 2.
Data Sharing Statement
