# Supplementary material for: Food Insecurity Prevalence and Risk Factors at a Large Academic Medical Center in Michigan
Source: JAMA Netw Open. 2024 Mar 26;7(3):e243723. doi: 10.1001/jamanetworkopen.2024.3723 (PMC10966414; doi:10.1001/jamanetworkopen.2024.3723)
Supplement: Supplement 1. — eTable 1. Domains and Questions Included in the Social Determinants of Health Screening at Michigan Medicine eTable 2. Demographic, Health, and Social Predictors of Interest in Assistance Among Adult Primary Care Patients Who Screened Positive for Food Insecurity [file jamanetwopen-e243723-s001.pdf]

Supplemental Online Content

Leung CW, Patel MR, Miller M, et al. Food insecurity prevalence and risk factors at a large academic medical center in Michigan. *JAMA Netw Open*. 2024;7(3):e243723. doi:10.1001/jamanetworkopen.2024.3723

eTable 1. Domains and Questions Included in the Social Determinants of Health Screening at Michigan Medicine
eTable 2. Demographic, Health, and Social Predictors of Interest in Assistance Among Adult Primary Care Patients Who Screened Positive for Food Insecurity

This supplemental material has been provided by the authors to give readers additional information about their work.

© 2024 Leung CW et al. *JAMA Network Open.*

eTable 1: Domains and Questions Included in the Social Determinants of Health Screening at Michigan Medicine

Domain	Question
Food	Within the past 12 months, you worried that your food would run out before you got money to buy more.
Food	Within the past 12 months, the food you bought just didn't last and you didn't have money to get more.
Housing instability	In the next 2 months, are you worried that you may not have stable housing?
Employment insecurity	Do you have a hard time finding work or another steady source of income?
Utility insecurity	In the last 12 months, has the utility company shut off your service for not paying your bills?
Medical care insecurity	In the last 12 months, did you not see a doctor when you needed or skip medications to save money?
Medication non-adherence	In the last 12 months, did you skip medications to save money?
Lack of transport kept you from medical care	In the past 12 months, has lack of transportation kept you from medical appointments or from getting medications?
Lack of transport kept you from work or daily living activities	In the past 12 months, has lack of transportation kept you from meetings, work, or getting things needed for daily living?
Child care or elder problems	In the last 4 weeks, did getting child care, elder care, or care for another person make it difficult to work, study, or get to medical visits?
Felt unsafe in home	Within the last year, have you been afraid of your partner or ex-partner?
Physical abuse	Within the last year, have you been kicked, hit, slapped, or otherwise physically hurt by your partner or ex-partner?
Emotional abuse	Within the last year, have you been humiliated or emotionally abused in other ways by your partner or ex-partner?
Rape/ sexual abuse	Within the last year, have you been raped or forced to have any kind of sexual activity by your partner or ex-partner?
Social isolation	Within the last 12 months, how often do you feel isolated from others?
Assistance	Do you want to get connected with resources for any of the above responses?

© 2024 Leung CW et al. *JAMA Network Open.*

eTable 2: Demographic, Health, and Social Predictors of Interest in Assistance Among Adult Primary Care Patients Who Screened Positive for Food Insecurity

Characteristic	Model 3	
	aOR[a]	95% CI
Age		
18-34	Ref.	
35-44	1.02	0.74, 1.41
45-54	1.01	0.74, 1.38
55-64	0.92	0.66, 1.26
65-74	0.81	0.54, 1.22
75 and over	0.87	0.50, 1.50
Sex		
Female	Ref.	
Male	0.75	0.60, 0.94
Race/ethnicity		
Hispanic	1.38	0.87, 2.19
Non-Hispanic Asian	1.48	0.76, 2.91
Non-Hispanic Black	2.08	1.65, 2.62
Non-Hispanic White	Ref.	
Other (Non-Hispanic)	1.31	0.81, 2.13
Marital status		
Married/ Significant Other	Ref.	
Single	1.19	0.93, 1.52
Divorced/ Widowed	1.63	1.20, 2.22
Health insurance type		
Private	Ref.	
Public	1.1	0.84, 1.45
Number of social needs		
0	Ref.	
1-2	3.62	2.57, 5.09
≥3	11.38	8.06, 16.06
Smoking		
Non-smoker	Ref.	
Current smoker	1.39	1.07, 1.81
Former smoker	1.18	0.93, 1.51
Alcohol use		
Non-drinker	Ref.	
Current drinker	0.71	0.57, 0.89
Former drinker	0.86	0.64, 1.16
BMI (in kg/m^2)		
<25.0	0.95	0.70, 1.28
25-29.9	Ref.	
30-34.9	1.26	0.94, 1.70
≥35.0	1.08	0.81, 1.42

aOR, Adjusted Odds Ratio

[a] Estimates are from multivariable logistic regression models

© 2024 Leung CW et al. *JAMA Network Open.*
