# Supplementary material for: Food Insecurity Prevalence and Risk Factors at a Large Academic Medical Center in Michigan
Source: JAMA Netw Open. 2024 Mar 26;7(3):e243723. doi: 10.1001/jamanetworkopen.2024.3723 (PMC10966414; doi:10.1001/jamanetworkopen.2024.3723)
Supplement: Supplement 2. — Data Sharing Statement [file jamanetwopen-e243723-s002.pdf]

Data Sharing Statement

Leung. Food Insecurity Prevalence and Risk Factors at a Large Academic Medical Center in Michigan. *JAMA Netw Open*. Published March 26, 2024.
doi:10.1001/jamanetworkopen.2024.3723

Data
Data available: No

Additional Information
Explanation for why data not available: The data contain PHI from electronic health records.
